# Contribution of intraoperative parathyroid hormone monitoring to the surgical success in minimal invasive parathyroidectomy

**DOI:** 10.3389/fsurg.2022.1024350

**Published:** 2022-09-21

**Authors:** Ismail Ethem Akgün, Mehmet Taner Ünlü, Nurcihan Aygun, Mehmet Kostek, Mehmet Uludag

**Affiliations:** Department of General Surgery, Şişli Hamidiye Etfal Education and Research Hospital, Istanbul, Turkey

**Keywords:** minimal invasive parathyroidectomy, primary hyperparathyroidism, parathyroid hormone, parathyroid - adenoma, parathyroid hyperplasia

## Abstract

**Background:**

The contribution of intraoperative parathyroid hormone monitoring to minimally invasive parathyroidectomy remains controversial. We aimed to evaluate whether intraoperative parathyroid hormone monitoring monitoring could contribute to minimally invasive parathyroidectomy in these patients.

**Methods:**

The data of the patients whose preoperative ultrasonography and technetium-99 m sestamibi scintigraphy imagings were positive and concordant for one gland and who underwent minimally invasive parathyroidectomy between 2003 and 2018 in our clinic, were evaluated retrospectively. Blood samples were collected at pre-excisional period, and at post-excisional 10 and 20 min; the intaoperative parathyroid hormone was measured, and the surgery was terminated without waiting for the result. Patients were divided into 2 groups according to the postoperative results, as those with normocalcemia (Group 1) and those with persistence (Group 2).

**Results:**

There were 195 patients in Group 1 and 14 patients in Group 2. The cure rate at the first surgery was 93.3%. Cure was achieved after the second operation in all patients in Group 2. Recurrent disease developed in 1 patient in group 1 and the overall cure rate was 99.5%. If intraoperative parathyroid hormone had been evaluated, cure could have been achieved at the first surgery with additional exploration, in 10 (71.4%) of 14 patients according to the insufficient decrease in parathyroid hormone value at the 10 min in Group 2, and in 9 (64.3%) of 14 patients according to the parathyroid hormone value at 20 min. However, due to insufficient decrease (false negative) in the parathyroid hormone value at the 10 and 20 min the rate of false negatives and unnecessary exploration would be 9.5% and 2.5%, respectively. With additional exploration, the cure rate in the first surgery could be increased by 4.3%–97.6% according to the 20 min intraoperative parathyroid hormone value.

**Conclusion:**

The cure rate in minimally invasive parathyroidectomy can be increased by minimizing unnecessary conversion to bilateral neck exploration, by evaluating intraoperative parathyroid hormone at 10 min in patients with positive and concordant scans, and intraoperative parathyroid hormone at 20 min in patients with inadequate decrease at 10 min intraoperative parathyroid hormone.

## Introduction

The only curative treatment of primary hyperparathyroidism (pHPT), which is a common endocrine disease, is surgery and the treatment should include resection of all enlarged parathyroid glands (1, 2). Pathological conditions causing pHPT are single adenoma (80%–85%), double adenoma (4%–5%), multiglandular hyperplasia (10%–15%) and parathyroid cancer (1%) (3). Whilst bilateral neck exploration (BNE) is still the gold standard operative approach in pHPT, minimally invasive parathyroidectomy (MIP) methods have become the standard treatment option in selected patients with positive imaging, due to the fact that the pathological cause is single gland disease in most of the patients and the increased availability of preoperative localization studies (2, 4).

Intraoperative monitoring of parathyroid hormone (ioPTH) in parathyroidectomy is an adjunctive method used to confirm cure or to detect whether there is still hyperfunctional residual parathyroid tissue after the pathological gland has been removed. It has been shown that ioPTH contributes to MIP, increasing the cure rate and reducing the need for reoperation (5, 6).

However, the contribution of ioPTH monitoring to MIP in patients with positive and concordant preoperative ultrasonography (USG) and scintigraphy is still one of the most debatable issue. Some studies have reported that ioPTH may have an additional contribution due to unexpected intraoperative findings in patients with concordant scans (7–9). In most of the studies, it has been reported that ioPTH does not increase the cure rate of MIP and is not necessary in patients with concordant scans (10–12). In patients with concordant scans, ioPTH may increase operative time, cost, and even the conversion rate due to false-negative results due to insufficient decrease in ioPTH (13–15).

Multi-gland disease is one of the major challenging factors for persistent disease (16). Currently, there is still no preoperative modality that can reliably diagnose multi glandular disease alone (17).

The contribution of ioPTH increases as the incidence of multiple gland disease increases (6). Although the rate of single gland disease has been reported as 99% in cases where MIBI scan and USG are concordant and a single gland is localized, in some studies it has been reported that multiglandular disease rate may increase to 10%–14% in these patients (18–20). This situtation suggests that intraoperative PTH measurement may contribute to MIP. However, the number of studies reporting that ioPTH increases surgical success is limited.

In this study, we aimed to evaluate whether ioPTH monitoring in patients with 2 concordant imaging modalities had an additional contribution to operation success in MIP.

## Materials and methods

The data of the patients operated with the diagnosis of pHPT in our clinic between 2003 and 2018 were evaluated retrospectively. Our center is a tertiary reference center for endocrine surgery. Cases in this study were selected patients as specified in the inclusion criterias. Before 2020, general surgery clinics in our hospital were divided into three separate clinics as 1th, 2nd and 3rd surgery clinics. During this period, 2 surgeons experienced in endocrine surgery were performing parathyroid surgery in our hospital. These 2 surgeons were working in 2 separate surgery clinics. The cases in this study were surgeries performed by a single experienced endocrine surgeon (MU). In this period, the rate of total primary HPT operation by this surgeon (MU) is over 500. In addition, the number of patients exceeds 650 with persistent patients referred from external centers, patients with familial HPT syndrome, surgeries performed for secondary and tertiary HPT. The other surgeon had a total of about 400 parathyroid surgeries, and his cases could not be included in the study.

Demographic, clinical, anatomical and operative data of all patients have been recorded in the clinical standard database in detail, and informed consents have been obtained from the patients for data collection.

Approval was obtained from the local ethics committee and patients’ data were analyzed according to the guidelines in the Helsinki Declaration.

All patients had a preoperative biochemical diagnosis. All patients with pHPT underwent technetium-99m-sestamibi (MIBI) single photon computed tomography (SPECT) parathyroid scintigraphy and USG.

Our hospital had only a SPECT scintigraphy device until 2018. In our clinic, USG and SPECT scintigraphy were routinely applied in patients with prHPT operation indication, and no additional imaging methods could have been applied, in that period. Until that date, the patients had SPECT scintigraphy. After the SPECT/CT device had been installed in 2018, routine preop SPECT/CT was started.

All patients are discussed in the endocrine council (including surgeon, endocrinologist, nuclear medicine specialist and radiologist) with preoperative laboratory and imaging methods.

In persistent patients, the initial imaging methods, surgical findings, and pathology results are reevaluated. Generally, USG and SPECT scintigraphy were applied in the patients in this study. SPECT/CT examination had performed in an external center before the second operation in 3 patients who were persistent. In this period, methods such as PET cholin and 4D CT could not have been applied.

Patients with a single gland detected with concordant preoperative USG and parathyroid MIBI SPECT scintigraphy, and whom were planned for focused surgery and who had a follow-up of at least 6 months, were included in the study. Also only patients underwent minimal invasive parathyroidectomy were included in this study. Patients with negative scan, discordant imaging, familial pHPT, patients with multiple endocrine neoplasia, patients who underwent reoperation in our center for persistent or recurrent pHPT whose first surgery was performed in another center, patients who underwent parathyroidectomy with standard cervical exploration and bilateral exploration, were excluded from the study. In addition, patients who underwent lobectomy with parathyroidectomy in the same operatipn were excluded from the study due to 2 glands were explorated in exploration area.

Parathyroidectomy: In our clinic, parathyroidectomy (bilateral exploration or MIP) has been performed under general anesthesia with the guidance of intraoperative nerve monitoring since 2012. An open MIP (minimal invasive parathyroidectomy) was performed with a 2.5–3 cm incision placed laterally (till the anterior edge of the sternocleidomastoid muscle) in patients with positive and concordant USG and MIBI SPECT scans. In patients with enlarged parathyroid glands in a localization compatible with imaging methods, the surgery was terminated after removal of the pathological gland. If enlarged parathyroid glands were not found in the localization defined in preoperative imaging methods, if a second enlarged parathyroid gland was found on the same side, or if the operative findings were not compatible with preoperative imaging methods, the incision was converted to a standard Kocher incision, and BNE was performed.

Frozen examination: Frozen examination is applied in parathyroid surgery in selected patients when necessary in our clinic. However, frozen examination was not routinely performed in patients who underwent MIP. If there is no doubt that the removed gland is enlarged parathyroid gland, frozen examination was not performed. Frozen examination was performed in selected patients in suspicious cases.

Intraoperative PTH measurement: In all patients, a blood sample was taken from the peripheral vein for PTH measurement at pre-excisional and at post-excisional 10 and 20 min, and the operation was terminated without waiting for the results.

Intraoperative quick PTH monitoring is not performed in our center; intact PTH assay is analyzed by the electrochemiluminescence immunoassay (ECLIA) method with the Cobas 8,000 (Roche Diagnostics GmbH, Mannheim, Germany) device in the central laboratory, the analysis takes approximately 25–30 min and the results are expressed in nanogram/L (pmol/L) (Ref: 15–65 ng/L (1.6–6.9 pmol/L).

Evaluation of ioPTH: The results were analyzed postoperatively to evaluate surgical success and these results were taken into account for the patient's discharge. For surgical success, the PTH value at the 10 or 20 min was considered sufficient if it fell below 50% of the pre-excisional value whileit was considered insufficient if there was less than 50% reduction, and the patients were investigated early for persistent disease.

Achieving and maintaining postoperative normocalcemia for 6 months was defined as surgical success in MIP, and recurrence of hypercalcemia within 6 months was defined as surgical failure. The accuracy of ioPTH was calculated based on the definitions followed: It was defined as true positive (TP) if there was a decrease of more than 50% in ioPTH at 10 and 20 min and the surgical success was predicted correctly; as false positive (FP) if the surgical success was not predicted correctly (if there was surgical failure); as true negative (TN) if the decrease was less than 50% and it predicted surgical failure correctly; and as false negative (FN) if it did not predict surgical failure correctly (if there was surgical success).

Recurrence of hypercalcemia within 6 months was defined as persistent disease, and presence of hypercalcemia after 6 months of normocalcemia period was defined as recurrent disease (1).

The patients were divided into 2 groups as those with normocalcemia after the first surgery (Group 1) and those with persistent hypercalcemia. (Group 2).

Calcium and PTH values were checked in all patients on the 1st postoperative day, and normocalcemic patients were discharged. If there was not enough PTH decrease in the postoperative evaluation of ioPTH values and/or hypercalcemia persisted, these patients were evaluated for parathyroid pathologies with additional imaging methods in the early period, and a surgical strategy was planned accordingly.

The patients were followed up in the outpatient clinic at 1st, 3rd, and 6th months postoperatively. PTH measurement and operation costs were calculated according to Turkish healthcare state hospital price policies.

### Statistical analysis

Statistical analysis of all data was performed using SPSS (Statistical Packages for the Social Sciences, software, edition 21, SPSS Inc. Chicago, USA).

Number and percentage for categorical variables; for numerical variables, mean, standard deviation were given. In the comparison of the groups, differences between the ratios of categorical variables were made with the Pearson Chi-square test or Fisher's Exact Test, and non-parametric comparisons were made with the Mann–Whitney *U* test. *P* < 0.05 was accepted statistically significant.

Calculations were made with the formulas defined for the ioPTH at 10 and 20 min: Sensitivity [TP/(TP + FN)], specificity [TN/(FP + TN)], positive predictive value (PPV) [TP/(TP + FP)], negative predictive value (NPV) [TN/(FN + TN)], accuracy [(TP + TN)/(TP + TN + FP + FN)].

## Results

A total of 210 patients who met the inclusion criteria were included in the study. There were 196 (39M, 157F) patients in Group 1 and 14 (3M, 11 F) patients in Group 2, and the general characteristics of the groups are summarized in Table 1. Preoperative Mg (1.97 + 0.24 vs. 1.77 + 0.41; *p* = 0.043), ALP (141 + 213 vs. 79 + 24; *p* = 0.040), 25-OH Vitamin D (Vit D3) (17.2 + 11 .8 vs. 9.6 + 11.6; *p* = 0.027) values were higher in Group 1 than in Group 2, respectively. In accordance with Endocrine Society clinical practice guideline; 25-OH Vitamin D (Vit D3) insufficiency was defined as the values between 21 and 29 ng/ml (525–725 nmol/liter) and the deficiency was defined as the value of <20 ng/ml (50 nmol/liter) in this study (21). Parathyroid pathology was a single adenoma in 196 patients (93.3%), double adenomas in 11 patients (5.2%), and hyperplasia in 3 patients (1.5%). Single adenoma was 99% vs. 7.2% in Group 1 and Group 2; double adenoma was 0.5% vs. 71.4% and hyperplasia was 0% vs. 21.4%, respectively, and the differences were significant (*p* < 0.001). The diameter and volume of the largest gland were evaluated in patients with double adenoma and hyperplasia (Table 1).

**Table 1:** Characteristics of patients with postoperative cure at first surgery (group 1) and of persistent cases (group 2) (PTH, parathyroid hormone; Ca, calcium; Mg, magnesium; P, phosphorus, 25(OH)vitD3: 25-hydroxyvitamin D3; ALP, alkaline phosphatase) (in accordance with endocrine society clinical practice guideline; 25-OH Vitamin D (Vit D3) insufficiency was defined as the values between 21 and 29 ng/ml (525–725 nmol/liter) and the deficiency was defined as the value of <20 ng/ml (50 nmol/liter) in this study (21).

	Group 1, *n*: 196	Group 2, *n*: 14	*p*
Age (mean + SD/year)	53 ± 14	55 ± 15	0.665
Gender (F/M) *n* (%)	157/39	11/3	1
Preop creatinine (mean + SD) (mg/dl) (0.5–0.9)	1.12 ± 0.21	1.09 ± 0.19	0.145
Preop PTH (mean + SD) (ng/L) (15–65)	325 ± 423	211 ± 92	0.971
Preop Ca (mean + SD) (mg/dl) (8,6–10)	11.3 ± 2.1	11.6 ± 0.7	0.299
Preop Mg (mean + SD) (mg/dl) (1.6–2.6)	1.97 ± 0.24	1.77 ± 0.41	0.043
Preop P (mean + SD) (mg/dl) (2.5–4.5)	2.59 ± 0.59	2.26 ± 0.55	0.057
Preop ALP (mean + SD) (mg/dl) (35–104)	141 ± 213	79 ± 24	0.040
Preop 25 (OH)vitD3 (Insufficiency: 21–29 ng/ml (525–725 nmol/liter) Deficiency: <20 ng/ml (50 nmol/liter))	17.2 ± 11.8	9.6 ± 5.8	0.027
Adenoma diameter (mean ± SD) (cm)	2.2 ± 1	2.3 ± 0.7	0.318
Adenoma volume (mean ± SD) (cm^3^)	2.7 ± 5.1	2 ± 3.7	0.204
Pathology *n* (%)			<0.01
Single adenoma	195 (99.5%)	2 (14.3%)	
Double adenoma	1 (0.5%)	10 (71.4%)	
Hyperplasia	0	2 (14.3%)	

In Group 1, the surgery was converted to BNE from MIP in 3 patients, double adenoma was detected in 1 patient, and single adenoma was detected in 2 patients. In one patient, 2 adenomas were found on the ipsilateral side and 2 normal parathyroids were seen in the contralateral side in BNE. In the other 2 patients, no pathological gland was found with MIP method and a single adenoma was found on the contralateral side in BNE. In these patients there was no technical difficulties for converting to the BNE. As it was described above, BNE was performed due to the presence of 2 pathological parathyroid glands in the ipsilateral region in one patient and the absence of pathological glands in the other 2 patients.

In Group 2, cure was achieved with the second surgical intervention in 6 of the 14 patients performed in the 1st week postoperatively, within 3 months in 5, and within 1.5 years postoperatively in 3 of them. In the persistent group, pathology was single adenoma in 2 patients (14.3%), double adenoma in 10 (71.4%) and hyperplasia in 2 (14.3%) In 2 patients, it was evaluated as intrathyroidal parathyroid adenoma due to intense involvement of the thyroid gland in the imaging methods before the first surgery. Lateral lobectomy was performed in these patients according to the MIP technique. In the frozen examination, it was reported that 2 lesions could have been intrathyroidal parathyroid gland. However, one of these lesions was reported as follicular carcinoma and the other as follicular adenoma after first surgery. After these lesions were removed, the second SPECT scintigraphy demonstrated that there was an involvement of the contralateral pathological gland. In these 2 patients, a single adenoma was removed in the first operation, single adenoma was removed in the second operation. (Frozen examination was false positive, the pathological gland was not removed in the first operation.). 10 patients had double adenomas and 2 patients had hyperplasia at the end of the second operation in the other 12 patients who had single adenoma in the first operation (Table 1).

The pathological parathyroid gland, which was the cause of the persistence, was in ectopic localization in 4 patients, in orthotopic position in 10 patients; and in 5 of these 10 patients, the pathological gland was on the contralateral side.

The cure rate at the first surgery was 93.3%. In persistent patients in Group 2, cure was achieved in the second operation. Recurrent disease developed in 1 patient in Group 1. The overall cure rate was 99.5%.

### How does ioPTH predicts surgical success?

TP, FP, TN, FN rates of 10 and 20 min IOPTH values are summarized in Table 2. (Table 2) 10 min ioPTH: Sensitivity for predicting surgical success was 89.8%, specificity 71.43%, PPV 97.78%, NPV 33.33% and accuracy 88.57% (Table 3). 20 min ioPTH: Sensitivity for predicting surgical success was 97.45%, specificity 64.29%, PPV 97.45%, NPV 64.29% and accuracy 95.24%, and sensitivity, NPV and accuracy were increased compared to 10 min values (Table 3).

**Table 2:** True positive, false positive, true negative, false negative rates of 10 and 20 min PTH in predicting surgical success (PTH, parathyroid hormone; TP, true positive; FP, false positive; TN, true negative; FN, false negative).

	TP *n* (%)	FP *n* (%)	TN *n* (%)	FN *n* (%)	Total (*n*)
10 min PTH					
Group 1	176 (89.8%)	0	0	20 (10.2%)	196
Group 2	0	4 (28.6%)	10 (71.4%)	0	14
Total	176 (83.8%)	4 (1.9%)	10 (4.8%)	20 (9.5%)	210
20 min PTH					
Group 1	191 (97.4%)	0	0	5 (2.6%)	196
Group 2	0	5 (35.7%)	9 (64.3%)		14
Total	191 (91%)	5 (2.4%)	9 (4.3%)	5 (2.4%)	210

**Table 3:** Sensitivity, specificity, PPV, NPV and accuracy rates of 10 and 20 min PTH values in predicting surgical success (CI, confidence interval; PPV, positive predictive value; NPV, negative predictive value; PTH, parathyroid hormone).

	Sensitivity % (95%CI)	Specifcity % (95%CI)	PPV % (95%CI)	NPV % (95%CI)	Accuracy % (95%CI)
10 min PTH	89.8 (84.68–93.65)	71.43 (41.9–91.61)	97.78 (95.05–99.02)	33.33 (22.72–45.96)	88.57 (83.47–92.54)
20 min PTH	97.45 (94.15–99.17)	64.29 (35.14–87.24)	97.45 (94.98–98.72)	64.29 (41.06–82.30)	95.24 (91.42–97.69)

### Possible contribution of ioPTH to the surgical success and the risk of unnecessary exploration

In 10 patients due to insufficient decrease in ioPTH at the 10 min, additional exploration could have provided cure and contributed 4.8% to the cure rate, increased the cure rate to 98.1%. In addition, due to insufficient decrease in 10 min ioPTH value in 20 patients (9.5% false-negative rate) it could have caused unnecessary additional exploration. By evaluating the 20 min PTH in patients with insufficient decrease in the 10 min PTH rate, the unnecessary exploration rate due to false negativity at 10 min ioPTH could be prevented in 15 patients, and the unnecessary exploration rate could have been reduced to 2.4%. However, if the 20 min PTH values of all patients were to be evaluated, 4 patients with false positive values would increase to 5 patients, and the cure rate could have reached 97.6% (Table 4).

**Table 4:** Possible contribution of 10 and 20 min IOPTH values to the surgical cure rate and the risk rates of unnecessary additional exploration (PTH, parathyroid hormone).

	Possible cure	Contribution to cure rate	Unnecessary exploration
10 min PTH	98.1%	4.8%	9.5%
20 min PTH	97.6%	4.3%	2.5%

### Cost

In our country, according to state hospital prices, cost of PTH measurement is approximately 1 dollar, and parathyroid adenoma surgery cost is 210 dollars. The cost of 3 ioPTH measurements is 1/70 of the cost of 1 parathyroid surgery. Costs such as IONM cost and additional radiological examination costs applied in persistent patients are not included in the price of the surgery. The cost of PTH measurements in our 210 patients were equal to the total cost of 3 operations. According to 20 min PTH values, 9 persistent diseases and secondary operations could have been prevented by further exploration. In our series, a minimum of 6 surgery costs could have been saved.

## Discussion

Today, with MIP in parathyroid surgery, recurrence, persistence, reoperation and high cure rates can be achieved similar to the rates achieved with BNE with a lower complication rate and shorter operation time (22). In addition to the developments in imaging methods, ioPTH measurement has also a critical role in the development of MIP. In a recent meta-analysis, patients who underwent MIP with ioPTH had a higher cure rate (OR, 3.88; 95%CI, 2.12–7.10; *p* < .001), and the need for reoperation was lower compared to MIP without ioPTH (OR, 0.40; 95%CI, 0.19–0.86; *p* = .02) (5). In these patients, it is the more common accepted opinion that ioPTH may not have a significant contribution or that it has a small contribution (23, 24). Our method in these patients supported this suggestion, too. Although blood samples were taken for ioPTH at pre-excisional, postexcisional 10 and 20 min in patients whose preoperative USG and SPECT scintigraphy were compatible and detected one gland, we terminated the operation without waiting for PTH results and evaluated the results in the postoperative period. In this study, we aimed to retrospectively evaluate how it would contribute to our current surgical results if we evaluated the results of PTH intraoperatively.

In our center, blood samples were taken for PTH at the pre-excisionaland post-excisional 10 and 20 min in all parathyroid operations, and PTH measurements were performed in the central laboratory. Although blood samples were taken for ioPTH in patients whose preoperative scans were concordant and showed a single gland, the operation was terminated without waiting for PTH results in order not to prolong the operation time and the results were evaluated in the postoperative period.

According to our results, the success rate of surgery without ioPTH was 93.3% and the surgical success rate could be increased with the monitoring of ioPTH in patients in whom USG and SPECT scintigraphy were concordant and localized to one gland. In these patients, the surgical success rate could be increased by 4.3%–97.6% with a decrease in the 10 or 20 min PTH level below 50%. In these patients, the surgical success rate could be increased by 4.3%–97.6% with a decrease in the 10 or 20 min PTH level below 50%. Our study demonstrated that ioPTH is cost-effective, and persistent disease operation costs that can be prevented with ioPTH are more than 3 times the total ioPTH monitoring costs in the entire study. The results of this study were that ioPTH monitoring could contribute to surgical success in these patients. According to these results, we have changed our surgical management after 2018. If the postexcisional 10 min PTH value decreased below 50% according to the pre-excisional PTH value, the operation was terminated. If there was not such a decrease below %50 in postexcisional 10 min PTH, the postexcisional 20 min PTH result was waited. If there was less than 50% decrease in both postexcisional 10 and 20 min than the bilateral exploration was started.

Bobanga and Mchenry reported that PPV was 94% in detecting a single abnormal parathyroid gland in patients with concordant Tc99m-SPECT scintigraphy and USG, and the preoperative imagings were different from the intraoperative findings in 6% of the cases. They concluded that 100% cure could be achieved with 10 min post-excisional ioPTH, and ioPTH is necessary to ensure cure in hyperparathyroidism and confirm abnormal parathyroid gland in patients with preoperative concordant imaging (7). Barczynski et al. reported that ioPTH may increase the cure rate by 18.9% in patients with single positive imaging and by 3.8% in patients with two positive concordant imaging; however, for maximum patient safety and surgeon's confidence, they strongly suggested ioPTH monitoring with MIP in all patients including patients with 2 positive imagings (8).

Stalberg et al. performed focused surgery in MIBI SPECT positive patients with predicted single gland disease, and measured ioPTH at preoperatively, pre-excisionnally, and at the 10 and 30 min post-excisionally, and the results were evaluated at the end of the operation similar to our study. In the study, the cure rate was found to be 98%, and it was reported that ioPTH results may actually contribute only by 1% to the decision-making in MIP, however, it may cause unnecessary exploration of 9% due to a decrease of less than 50% in the 10 min ioPTH, and that a high rate of cure can be achieved with MIP without ioPTH in many patients, whose lesions were localized with the preoperative imaging methods (12). On the other hand, Sartori et al., evaluated the contribution of ioPTH to the surgical cure rate comparing the focused surgery applied with or without ioPTH in patients with preoperative concordant USG and MIBI scintigraphy findings. They found similar cure rates (97.6% vs. 96.3%, *p* = 0.408, respectively) and stated that ioPTH did not improve the results in these patients (10).

In the experienced endocrine surgery center of Di Marco et al., the conversion rate to BNE was 16.3% in selected patients who underwent MIP (focused parathyroidectomy) without ioPTH and with 2 concordant imaging modalities in a large series, and the rate of multigland disease in patients who underwent conversion was 54.5%. When patients with focused parathyroidectomy were compared to the patients with BNE, the rates of persistent and recurrent diseases were similar (2.3% and 2.2%, *p* = 0.44, respectively), and the complication rates were found to be lower (1.2% vs. 3.2%, *p* < 0.01, respectively). The authors concluded that MIP without ioPTH can be performed on patients with concordant imaging with a selective approach, provided that conversion to BNE is made when necessary in experienced hands (25).

Therefore, it is recommended by some authors that ioPTH should be applied during MIP routinely, not only in selected cases (9, 20, 26). Also Medas et al. reported in their current meta-analysis that ioPTH reduces surgical failure even in patients in whom the pathological gland can be clearly localized and concordant with both preoperative imaging method; recommended that routine use of ioPTH should be included in primary hyperparathyroidism surgery in the new guidelines (6).

The major (most important) cause of persistent disease is multi-gland disease, and in our study, the rate of multi-gland disease was 6.7% in the series and 85.7% among persistent patients (group 2).

Despite all the developments in imaging methods, there is no single imaging method or combined imaging method that can predict preoperative single and multi-gland disease. In a meta-analysis of preoperative localization studies, the pooled sensitivity and PPV of USG were 76.1% (95% CI, 70.4%–81.4%) and 93.2% (95% CI, 90.7%–95.3%), respectively; and for MIBI SPECT, pooled sensitivity and PPV were 78.9% (95% CI, 64%–90.6%) and 90.7% (95% CI, 83.5%–96.0%), respectively (27).

In the study of Tunca et al., the sensitivity rates of USG and MIBI scintigraphy were 76% and 81%, respectively, and PPV of imaging were 90% and 91%, respectively. In patients with concordant USG and MIBI scintigraphy, the rate of uniglandular disease was 90%, the rate of multiglandular disease was 10%, and the total localization rate was 91%. Although PPV was 100% in these patients with concordant imaging, the sensitivity was 91% (19). In patients with multiglandular disease, USG and SPECT/CT were concordant for single gland in approximately 30% of patients and suggested a single gland disease (17).

The cost effectiveness of ioPTH monitoring in MIP is also among the topics of discussion. In a recent multicentric European study, it has been reported that ioPTH is not cost-effective in cases with positive and concordant scans, and may even increase the conversion rate by 2.7% due to false negativity (15).

Morris et al. examined 4,280 patients in 17 studies and applied a model to predict the cost effectiveness and probability of ioPTH in patients with localized pHPT. Inappropriate location and high reoperation cost are both independent factors that increase the additional value of ioPTH. If the cost of ioPTH in a case is less than $110, it reduces the total cost. They found that the ioPTH strategy may be cost effective if the rate of preoperatively undetected multiple gland disease was greater than 6%, or if the reoperation cost was more than $12,000 (primary surgery cost: $3,733). As a result, the contribution of ioPTH in this model depends on the institution-specific factors and causes an additional cost of approximately 4%, while its contribution to MIP is marginal, increasing the success rate from 96.3% to 98.8% (28).

Our institution is a state hospital, and in our country, the state healthcare insurance price (official social insurance price) of ioPTH is approximately 1.5% of the primary surgery price, being cost-effective. In our total of 210 cases, the sum of total ioPTH prices was equal to 3 primary parathyroid adenoma surgeries, and 9 persistent patients could have been prevented with the price of these measurements. Since the cost of the second preoperative examination and re-operation of these 9 patients is higher than that of the primary surgery, we think that it may contribute significantly to the decrease in the total price and is cost effective.

The criterias for ioPTH monitoring to predict surgical success are still controversial. There is a lack of standardization regarding baseline PTH value samples, percentage of decrease used as cut-off, time of measurement and sampling frequency (24). Since the PTH has an average half-life of 3–5 min, a decrease of more than 50% at post-excisional 10 min relative to the highest pre-excisional or pre-incisional value (Miami criteria) has the highest relevance for predicting intraoperative cure (29). If the 10 min ioPTH value does not decrease enough, in most centers, the surgery is converted to bilateral exploration, while an additional 20 min ioPTH measurement is recommended in some centers. Khan et al. stated that in patients with a PTH decrease of less than 50% at the 10 min, the PTH value at the 20 min decreased by more than 50% in 46% of the patients, thus preventing unnecessary BNE in these patients (18).

In this study, the sensitivity could be increased from 89.8% to 97.5%, and the suitability from 88.6% to 95.2%, compared to ioPTH measurement at 20 min compared to ioPTH measurement at 10 min in predicting surgical success. According to the PTH value at the 10 min, NPV increases from 33% to 64% and unnecessary BNE decreases by 75% from 9.5% to 2.4%. We think that this BNE rate is not a problem in experienced centers and will not occur a great risk.

Although the portable quick PTH measurement results are obtained in approximately 10 min in the operating room, the most important disadvantage is the average waiting time of 25–30 min in the PTH measurement in the central laboratory (30). However, the rapid PTH measurement in the operating room is not performed in many centers as in our center, and it is a more expensive method than the one checked in the central laboratory (31). Since our center is a government hospital, this waiting time during the operation does not significantly increase the overall cost of the operation. The sensitivity, specificity and accuracy of the ioPTH measurement method performed in the central laboratory are similar to the rapid PTH measurement method in the operating room and can be used as an alternative method (32).

The main limitations of this study were that it included single-center results, it was retrospective, and although ioPTH was obtained in all patients with concordant scans who underwent MIP, the results were evaluated postoperatively, and had no effect on the surgical strategy in these patients. However, the possible contributions of ioPTH to surgery were evaluated in the same patients.

As a result, ioPTH measurement can increase the cost-effectiveness and surgical cure rate in patients with positive and concordant USG and MIBI SPECT scintigraphy scans. In patients with insufficient ioPTH decrease at 10 min, checking ioPTH at 20 min may contribute to MIP and minimize the conversion rate to unnecessary BNE. With this protocol, in patients with positive and concordant USG and MIBI SPECT scintigraphy scans for one gland, the conversion to unnecessary BNE may be minimized, and the cure rate of 93.3% in MIP without ioPTH, may be increased to 97.6%.

## Data Availability

The original contributions presented in the study are included in the article/Supplementary Material, further inquiries can be directed to the corresponding author/s.
